# The role of glacial cycles in promoting genetic diversity in the Neotropics: the case of cloud forests during the Last Glacial Maximum

**DOI:** 10.1002/ece3.483

**Published:** 2013-01-25

**Authors:** Santiago Ramírez-Barahona, Luis E Eguiarte

**Affiliations:** Departamento de Ecología Evolutiva, Instituto de Ecología, Universidad Nacional Autónoma de MéxicoMexico City, Mexico

**Keywords:** Climate change, historical demography, Last Glacial Maximum, paleoecology, phylogeography, population genetics, refugia

## Abstract

The increasing aridity during the Last Glacial Maximum (LGM) has been proposed as a major factor affecting Neotropical species. The character and intensity of this change, however, remains the subject of ongoing debate. This review proposes an approach to test contrasting paleoecological hypotheses by way of their expected demographic and genetic effects on Neotropical cloud forest species. We reviewed 48 paleoecological records encompassing the LGM in the Neotropics. The records show contrasting evidence regarding the changes in precipitation during this period. Some regions remained fairly moist and others had a significantly reduced precipitation. Many paleoecological records within the same region show apparently conflicting evidence on precipitation and forest stability. From these data, we propose and outline two demographic/genetic scenarios for cloud forests species based on opposite precipitation regimes: the dry refugia and the moist forests hypotheses. We searched for studies dealing with the population genetic structure of cloud forest and other montane taxa and compared their results with the proposed models. To date, the few available molecular studies show insufficient genetic evidence on the predominance of glacial aridity in the Neotropics. In order to disentangle the climatic history of the Neotropics, the present study calls for a general multi-disciplinary approach to conduct future phylogeographic studies. Given the contradictory paleoecological information, population genetic data on Neotropical cloud forest species should be used to explicitly test the genetic consequences of competing paleoecological models.

## Introduction

The Pleistocene glacial periods have been regarded as major factors influencing the geographical distribution, demographic dynamics, and patterns of genetic diversity of species (Comes and Kadereit 1998; Haffer and Prance 2001; Hewitt 2004; Soltis et al. 2006; Stewart et al. 2010). Paleoecological and molecular studies have provided abundant data concerning the vegetation changes resulting from these glacial cycles (Hewitt 2004; Stewart et al. 2010). The main bulk of research has been focused on northern latitudes, particularly on taxa inhabiting Europe and North America (e.g., Comes and Kadereit 1998; Petit et al. 2003; Skrede et al. 2006; Soltis et al. 2006; Waltari et al. 2007; Wang et al. 2009). There is a consensus view that the advance of ice sheets and temperature descent through Last Glacial Maximum (LGM, ∼23–18 kyr BP) had a major impact on the distribution of species in these regions. Temperate species persisted through this period in isolated southern refugia where climatic conditions were less extreme (Hewitt 2004; Soltis et al. 2006; Provan and Bennett 2008). Thus, the cold glacial pulses have been regarded as main forces driving the divergence of populations.

The Neotropics is regarded as having an extremely diverse flora and fauna (Gentry 1982; Kier et al. 2009). Specifically, the mountainous regions have been considered as exceptionally rich centers of biodiversity and endemism (Kier et al. 2009). Given their complex topography and habitat heterogeneity, there is likely to be a high genetic diversity within most montane species and signals of the genetic and evolutionary process that occurred as a consequence of the Pleistocene glacial cycles (Haffer 1969; Gentry 1982; Jaramillo-Correa et al. 2008). There have been attempts to explicitly uncover the effects of these glacial periods on the genetic diversity of Neotropical montane species (e.g., Aguirre-Planter et al. 2000; Jaramillo-Correa et al. 2008; Koscinski et al. 2008; Carnaval et al. 2009; Hensen et al. 2011). In spite the availability of paleoecological data for many Neotropical regions, the available phylogeographic studies seldom use these data to generate explicit predictions on the genetic structure of extant populations. Thus, there is still considerable ambiguity regarding the effect of glacial periods on the genetic structure of Neotropical montane species.

Several authors have proposed the refugia theory as the underlying model for glacial and postglacial population dynamics of tropical species during the LGM (Haffer 1969; Van der Hammen and Hooghiemstra 2000; Haffer and Prance 2001; Mourguiart and Ledru 2003). The basic tenet of the tropical refugia theory is that the cooler glacial climates were also characterized by a drastic reduction in precipitation. The widespread aridity during glacial times caused tropical forests to be compressed, fragmented, and isolated into distinct forest patches separated by intervening non-forest vegetation (Haffer 1969; Toledo 1982; Carnaval and Moritz 2008; Carnaval et al. 2009; de Mello-Martins 2011).

Some authors have questioned the pervasiveness of this suggested aridity during the LGM and therefore the validity of the refugia model (Colinvaux et al. 2000; Colinvaux and de Oliveira 2001; Baker et al. 2003). These authors support Van der Hammen's (1961) inference that “the most important cold phases were wet phases at the same time and the warmer phases dry”. Contrary to the refugia scenario, the supporters of this view claim that precipitation was not significantly reduced during the LGM. Consequently, tropical forests had stable and continuous distribution during glacial periods (Farrera et al. 1999; Hostetler and Mix 1999; Caballero et al. 2010).

The cloud forests are a suitable system to investigate the direction of change in precipitation during the LGM and its effects on tropical montane plant species because they are extremely vulnerable to changes in the hydrological cycle (Foster 2001). The cloud forests are characterized by persistent cloud immersion, which directly influences the moisture availability by a constant input of large amounts of water as horizontal precipitation (Foster 2001). The cloud forests are some of the most diverse Neotropical forest communities, characterized by the considerable number and proportion (10–30%) of endemic species (Gentry 1982; Foster 2001; Luna-Vega and Magallón 2010).

Most of the proposed refugia for Neotropical lowland species lie within mountain regions that are currently cloud forest areas (Prance 1982). This is the direct result of aridity displacing forest species into mid-elevations where the humidity would be maintained better that in the lowlands. Paleoecological data indicate that Neotropical cloud forests were subjected to down-slope migration in response to glacial cooling (Colinvaux et al. 1996; Urrego et al. 2005; Caballero et al. 2010; Valencia et al. 2010). Thus, under cool-arid conditions cloud forests would have been subjected to opposing forces and consequently be compressed into distinct refugia (Hooghiemstra and Van der Hammen 2004). Given the debate on glacial aridity, it remains unsubstantiated whether cloud forests were contracted or not into forest refugia during the LGM.

The knowledge on precipitation during the LGM, and hence on the distribution of Neotropical forests, is largely based on limited and conflicting paleoecological data. In this sense, this review presents an approach to test the proposal of widespread glacial aridity by way of its expected genetic effects on cloud forest species. To achieve this, in the first section we present a review of several paleoecological studies representing 48 records throughout tropical America. Based on these data, in the second section we propose two demographic scenarios for cloud forests based on opposite precipitation regimes: the dry refugia and the moist forests hypotheses. Then we outline the basic genetic consequences of these models as expectations for the glacial dynamics of cloud forest species. Finally, within the framework of these criteria, in the last section we present a brief overview of some molecular studies available for cloud forest species and other montane taxa.

## Literature search

**Figure 1:**
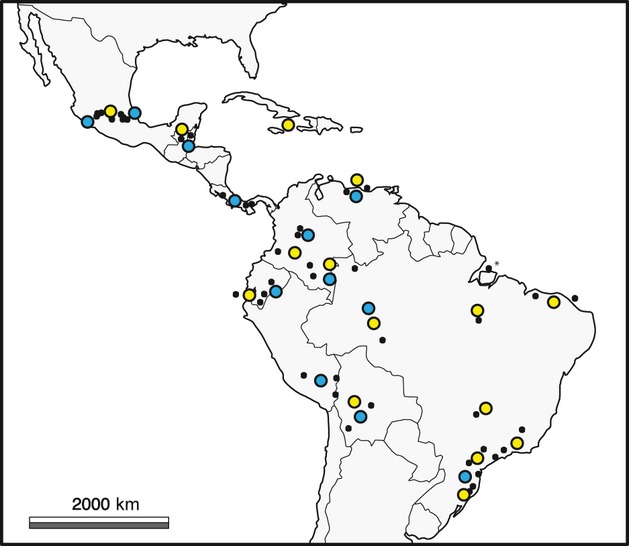
Map of the Neotropics with the geographical location of the paleoecological records reviewed (black dots) and the inferred humidity conditions during the LGM (colored circles). Blue and yellow circles represent moist and dry conditions, respectively.

In the first section, we focused our survey on paleoecological data encompassing the LGM for the Neotropics. To achieve this, we performed a reference search on key articles on the topic (e.g., Van der Hammen 1961, 1974; Haffer 1969; Prance 1982; Bush and Colinvaux 1990; Bradbury 1997). The final review included studies covering 48 paleoecological sites throughout the Neotropics (Fig. 1).

In the last section, we searched for relevant articles dealing with the intra-specific phylogeography of cloud forest taxa on the Web of Knowledge database (http://apps.webofknowledge.com), using the following search terms combinations: cloud forests* AND phylogeography*. This resulted in only two studies dealing with plant species, both in Mexico and upper Central America. Given the paucity of information, we included other relevant studies for Neotropical montane forest taxa in the discussion.

## The LGM in the Neotropics

In his seminal work of 1961, Van der Hammen made two basic inferences about climate change in the Neotropics during the LGM. The first inference on the nature of temperature descent, along with the subsequent down-slope displacement of montane forests, has been widely accepted. On the contrary, there is an ongoing debate surrounding Van der Hammen's second inference that the most important cold periods were characterized by increased humidity and that warmer periods were dry. In this context, the next section addresses the main inferences on precipitation during the LGM drawn from paleoecological data in tropical America (Table 1; Fig. 1).

**Table 1:** Main observations and inferred humidity conditions during the LGM for Neotropical regions based on several paleoecological records. Only references to the most relevant papers are included for each region (see text for an extended reference list)

Region	Humidity conditions	Authors
Central Mexico	Dry	Caballero et al. (2010)
Upper Central America	Dry	Anselmetti et al. (2006)
Upper Central America	Moist	Mueller et al. (2010)
Lower Central America	Moist	Colinvaux et al. (1996)
Northern Andes	Moist	Valencia et al. (2010)
Northern Andes	Dry	Mourguiart and Ledru (2003)
Amazonia	Dry	Van der Hammen and Hooghiemstra (2000)
Amazonia	Moist	Colinvaux et al. (2000)
SE Brazil	Dry	Behling (2002)

### Mexico, Central America, and the Caribbean

Caballero et al. (2010) recorded a 1000–1500 m lowering of glaciers in Central Mexico. From this, the authors interpreted an 8°C cooling. The glacier descent was less pronounced in Central Mexico than near the coasts. This suggests that Central Mexico was dryer than the Pacific and Gulf coasts. Accordingly, the dominance of grass pollen in the records and reduced lake levels reflect an overall cool and dry climate. This climate favored the down-slope migration of montane forests species (e.g., *Pinus, Quercus*, *Alnus*). Many of these taxa, particularly those from cloud forests, probably inhabited areas of wet microclimates immersed in a matrix of dry conditions (Caballero et al. 2010).

Moreover, the studies of Leyden et al. (1993), Bradbury (1997), Anselmetti et al. (2006), and Metcalfe et al. (2009) suggested a reduction in wet season precipitation for upper Central America and Mexico. Bradbury (1997) observed dominant grasslands in records around the Caribbean basin, indicative of cooler and dryer climate. The dominance of pollen from *Pinus, Cupressus*, *Juniperus, Myrica,* and Poaceae is taken as evidence of enhanced dry conditions (Leyden et al. 1993; Bradbury 1997). Accordingly, Anselmetti et al. (2006) observed an 87% reduction in the water volume of Lake Peten-Itza, Guatemala. This reduction has also been observed in New River Lagoon, Belize (Metcalfe et al. 2009) and Lake Quexil, Guatemala (Leyden et al. 1993).

It has been suggested that the summer precipitation regime was collapsed, resulting in arid conditions extending over much of Mexico, Central America, and the Caribbean (Hostetler and Mix 1999; Metcalfe et al. 2000). However, Metcalfe et al. (2000) suggested that Central Mexico remained cool and moist. According to the authors, winter precipitation was enhanced and evaporation was reduced by the southward position of the Laurentide ice sheet. This would have resulted in less seasonality and prevailing wet conditions even with reduced wet season precipitation (Hostetler and Mix 1999). In line with these observations, Hodell et al. (2008) and Mueller et al. (2010) suggested that there was no effective decrease in humidity in upper Central America. The records from Lake Peten-Itza showed a rich clay deposit and a pollen diagram dominated by *Pinus, Quercus,* and some mesic elements such as *Liquidambar* and *Alnus*. These records are indicative of a montane forest community thriving in lower elevations under cool and moist conditions (Hodell et al. 2008; Bush et al. 2009). The down-slope migration of montane taxa has also been observed in pollen records from Panama (Bush and Colinvaux 1990; Colinvaux et al. 1996). These authors showed that montane taxa (e.g., Ericaceae, *Quercus*, *Ilex, Thalictrum, Symplocus, Ranunculus, Magnolia, Alnus, Podocarpus, Weinmania*) were growing 500–800 m lower than their present range.

The analyses of González et al. (2008) from the Cariaco basin in northern Venezuela also support the lowering of montane forest taxa in response to cooling. These taxa, including cloud forest elements (e.g., *Ilex, Juglans, Podocarpus,* Cyatheaceae) increased in abundance at lower elevations over cooler periods, suggesting stable humidity conditions. However, González et al. (2008) point out that dry seasonal forests also showed an expansion during these periods. This last result led the authors to support a cool and dry climate extending throughout the Caribbean basin. The proposed reduced precipitation hindered the down-slope expansion of cloud forests, which were displaced into discrete areas with stable humidity conditions (González et al. 2008; Caballero et al. 2010).

### The Andes, Amazonia, and southeastern Brazil

As initially proposed by Van der Hammen (1961), montane forests in the Colombian Andes grew 1000–1500 m down-slope of their present range (Van der Hammen 1974; Bush et al. 1990; Paduano et al. 2003; Hooghiemstra and Van der Hammen 2004; Brunschön and Behling 2009; Hillyer et al. 2009; Valencia et al. 2010). The displacement of montane vegetation and the observed glacier descent of 500–900 m (Hostetler and Mix 1999) have been used to infer a 4.5°C cooling.

The montane forests around Lake Pacucha (southern Peru) were replaced by grassland vegetation (Valencia et al. 2010). Other records confirm the replacement of montane forests by grassland vegetation throughout the Andes (Bush et al. 1990; Behling et al. 1998; Baker et al. 2003). Accordingly, the records from Lake Consuelo (southern Peru), located 1700 m lower than Lake Pacucha, show the down-slope migration and expansion of montane taxa (Bush et al. 2004; Urrego et al. 2005). The vegetation around Lake Consuelo had upper montane taxa being dominant (e.g., *Alnus, Hedyosmum, Myrsine, Podocarpus, Symplocos, Vallea,* and *Weinmannia*), which were subsequently displaced upslope during the Holocene and are today largely absent around Lake Consuelo. Taken together, these records show an expansion of montane taxa into lower elevations during the LGM. This is taken as evidence of prevailing cool and moist conditions in the region.

The prevalence of montane forests throughout the Andes (i.e., Colombia, Ecuador, Peru, Bolivia) and the inferred moist conditions have been confirmed by the high percentage of fresh water diatoms and low levels of carbonates in sediment cores from Lake Titicaca, Peru (Baker et al. 2001a). These authors inferred that Lake Titicaca was deep, fresh, and overflowing. In this context, the oscillations between gypsum and clay layers at Salar de Uyuni (southern Bolivia) indicate that precipitation was not reduced in the Bolivian and Peruvian Andes during the LGM (Baker et al. 2001b).

In spite the stable precipitation inferred for the Andes, some records from lowland regions in Amazonia show dominant grasslands during the LGM, which were later replaced by modern rain forests (Van der Hammen 1974; Heusser and Shackleton 1994; Hooghiemstra and Van der Hammen 1998, 2004; Van der Hammen and Hooghiemstra 2000). These records support reduced precipitation and prevailing dry conditions throughout the lowlands of the Amazon basin. In accordance, Van der Hammen and Hooghiemstra (2000) suggested a 45% reduction in precipitation. In turn, Hooghiemstra and Van der Hammen (2004) suggested that the elevational range of montane forests was compressed as much as 50%. As expected, the low abundance of forest elements indicates that tropical forests contracted and were less continuous during this arid period (Heusser and Shackleton 1994; Behling et al. 1998; Hessler et al. 2010).

However, other records from lowland Amazonia do not show a replacement of rain forest by savanna-like vegetation. Colinvaux et al. (2000) and Colinvaux and de Oliveira (2001) showed that forest cover remained constant in the lowlands of western Amazonia, with significant incursions of montane taxa such as *Alnus, Podocarpus, Ilex,* and *Hedyosmum*. Accordingly, sedimentary records from the Amazon fan detect an increase of montane taxa during the LGM (Hoorn 1997). These records do not show an overrepresentation of grass pollen, which would otherwise be indicative of savanna-like vegetation expanding throughout Amazonia (Hoorn 1997; Colinvaux et al. 2000; Colinvaux and de Oliveira 2001).

In the highlands of southeastern Brazil there is evidence of the dominance of grassland taxa and of a poor representation of montane elements (Ledru et al. 1996; Behling 1997). The records also show the occurrence of montane forests species in the lowlands of the Brazilian Atlantic coast (Behling 1997, 2002). This altitudinal displacement implies a cooler than present climate. The extension of open grassland vegetation has been taken as evidence of aridity extending throughout the region (Behling 2002; Ledru et al. 2007; Hessler et al. 2010). Ledru (1993) and Ledru et al. (1996) inferred cooler and humid conditions before and after the LGM in central Brazil. These conditions are reflected in the dominance of montane forest taxa, such as *Araucaria, Podocarpus, Drymis,* and *Cyathea*, before and after this period. In addition, Ledru (1993) and Ledru et al. (1996) observed a sedimentary hiatus during the LGM, which is taken as evidence of extreme aridity. However, the same authors proposed that seasonality in precipitation was reduced as a result of the northward migration of the polar front, which would have augmented the humidity during the dry season.

## Summary of the paleoecological data during the LGM

From the above review we observe that precipitation changes during the LGM were not homogenous across the Neotropics. Some regions remained fairly moist (e.g., the Andes) and others had a significantly reduced precipitation (e.g., southeastern Brazil). This is not surprising given the complex nature of the atmospheric-oceanic circulation system over the Neotropics (Hessler et al. 2010). However, many paleoecological records within the same geographical region show apparently conflicting evidence on forest stability during the LGM, mainly resulting from contrasting interpretations of pollen and sedimentary sequences (Urrego et al. 2005). Furthermore, the conflicting evidence has been enhanced by differing ideas on the effect of reduced precipitation on the continuity of tropical forests. Some authors claim that the reduction in wet season precipitation has a minor effect on the continuity of forest cover compared with the overall change in precipitation seasonality (Colinvaux et al. 2000; Bush and Silman 2004; Bush and de Oliveira 2006).

## Cloud forests during the LGM

In the absence of conclusive paleoecological data, analyzing the genetic structure of cloud forest plant species can be used to assess the two models of precipitation. Given their high hydrological vulnerability, even the most conservative estimate of precipitation reduction would result in significant changes in the demography and distribution of these species. Molecular studies that explicitly test the genetic structure of cloud forests species can provide some insight into the climatic history of Neotropical forests and thus serve to further corroborate the paleoecological data.

**Figure 2:**
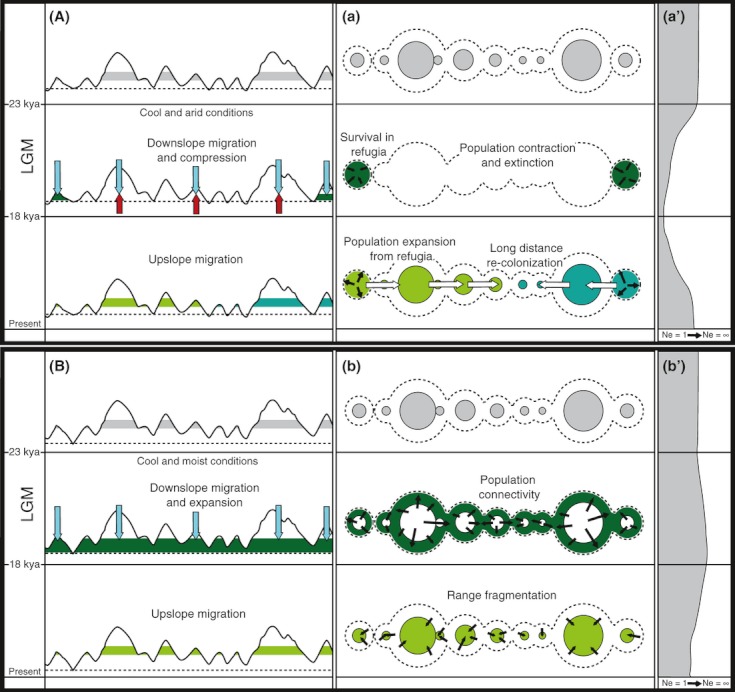
Models of the distributional and demographic dynamics of cloud forests during the Last Glacial Maximum (LGM, ca. 23–18 kya). (A) Dry refugia model. (B) Moist forests model. The distribution and abundance of cloud forests are represented in a cross-section (A, B), the corresponding aerial view (a, b) and a graphic of the idealized population change over time (a', b'). From top to bottom: the distribution of cloud forests before (gray), during (dark green), and after (light green/blue) the LGM. Blue and red arrows indicate the direction of displacement resulting from cooling and aridity, respectively (A, B). Black solid arrows indicate the direction and magnitude of population growth and white arrows indicate the direction of re-colonization (a, b). The dashed line represents minimum altitude level attained by the down-slope migration during the LGM.

The next section gives a brief description of the expected genetic consequences of the dry refugia hypothesis, which are subsequently compared with those expected under the moist forests model (Table 2; Fig. 2). The proposed consequences are largely based on the vast number of studies undertaken for temperate taxa (e.g., Bennett, 1985; Comes and Kadereit 1998; Haffer and Prance 2001; Knowles 2001; Hewitt 2004; Williams et al. 2004; Schönswetter et al. 2005; Soltis et al. 2006; Carstens and Knowles 2007; Bennett and Provan 2008; Holderegger and Thiel-Egenter 2009; Stewart et al. 2010).

**Table 2:** Demographic and genetic consequences for cloud forest taxa predicted by the two precipitation models for the Neotropics during the Last Glacial Maximum: the dry refugia and the moist forests models

Model	Demographic and genetic consequences
*Dry refugia*	
	Re-colonization and demographic expansion from small population sizes.
	Loss of genetic diversity and marked genetic structuring of populations.
	Species with concordant patterns of isolation and divergence between refugial lineages.
*Moist forests*	
	Range expansion and population connectivity in the lowlands.
	Upslope range fragmentation and little to no demographic expansion.
	Increased genetic diversity resulting from spatial heterogeneity and diffuse genetic structuring of populations.
	Less likely phylogeographic concordance across species.

**Figure 3:**
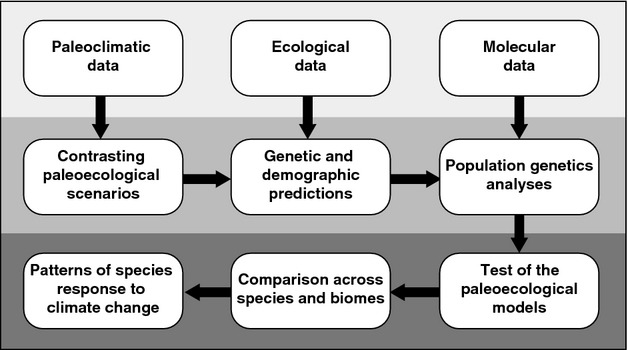
Proposed general framework for conducting phylogeographic studies on Neotropical species based on the integration of paleoclimatic, ecological, and molecular data.

### The dry refugia model

The dry refugia hypothesis was originally proposed and subsequently debated for lowland tropical forests, mainly in the Amazon (Haffer 1969). The present model is a direct extrapolation of this hypothesis, relying in the fact that the proposed refugia were located at mid-elevations in mountain regions with stable temperature and humidity conditions (Haffer 1969; Toledo 1982; Burnham and Graham 1999; Haffer and Prance 2001; Hooghiemstra and Van der Hammen 2004; Bush and de Oliveira 2006). Under this scenario, the cloud forests were displaced and compressed into refugia by the opposing effects of aridity and cooling. Subsequently, these populations would expand and re-colonize the species former range at the onset of more humid and warm conditions (Fig. 2).

Given the small size and isolation of the refugial populations, genetic drift would cause the average probability of identity by descent to increase within these populations. This would be reflected in allele sorting and the fixation of different alleles in separate refugia (Vendramin et al. 2008; Sommer and Zachos 2009). Populations from different refugia would thus become differentiated from each other, accumulate new mutations and would likely show reciprocal monophyly (Petit et al. 2003; de Mello-Martins 2011). In addition, gene flow between isolated populations from different refugia, while possible, would be limited (Schoville et al. 2011). Extant lineages originated in separate refugia would be significantly divergent and therefore show significant genetic differentiation (Knowles 2001; Heuertz et al. 2004). Ultimately, the observed levels of genetic differentiation would be dependent on the extent of former refugia populations and on the levels of past gene flow between them (Soltis et al. 2006).

The demographic contraction and subsequent expansion away from refugial populations would have a marked effect on the frequency distribution of allele differences in extant populations (Rogers and Harpending 1992). After expansion, the distinct refugial lineages would consist of one or few common alleles and would accumulate an excess of rare alleles separated by few mutational steps. Thus, a unimodal distribution of allele differences (mismatch distribution) and a star-shaped allele genealogy are expected for expanding refugial lineages (Slatkin and Hudson 1991; Rogers and Harpending 1992).

The refugial scenario would also have profound effects on the geographical distribution of alleles. In general, the re-colonization out of refugia would involve repeated long-distance dispersal over relatively large geographical areas. Therefore, this process would be characterized by repeated founder effects and by the subsequent loss of allelic richness (Comps et al. 2001; Hewitt 2004; Excoffier et al. 2009). The occurrence of founder effects would be negatively associated with the dispersal ability of species (Petit et al. 1997, 2003; Lessa et al. 2003; Tremetsberger et al. 2003; Bialozyt et al. 2006; Soltis et al. 2006; Excoffier et al. 2009; Callens et al. 2011). Thus, depending on the dispersal ability of species and the time elapsed, the re-colonization out of refugia would result in the decline of genetic diversity away from refugial populations and in a high differentiation of the most recently colonized populations (Slatkin 1993; Austerlitz et al. 1997; Lessa et al. 2003; Tremetsberger et al. 2003; Heuertz et al. 2004; Hewitt 2004; Ehrich et al 2007). Additionally, as the common alleles present in refugia would be the more likely source of re-colonization, these would automatically become frequent in the founding populations and therefore would become widespread following colonization. On the other hand, the rare alleles would accumulate around refugial populations, resulting in a high probability of geographical clustering of these alleles (Comps et al. 2001; Hewitt 2004).

The loss of genetic diversity during re-colonization and, in many cases the secondary contact between different refugial lineages, would break down the pattern of isolation by distance even in species with dispersal mechanisms that should promote such a pattern (Slatkin 1993; Petit et al. 2003; Caetano et al. 2008; Provan and Bennett 2008; Sommer and Zachos 2009). However, the colonization dynamics out of single refugia could also result in isolation by distance because genetic differentiation reaches the equilibrium state of isolation by distance relatively quickly (Slatkin 1993).

Finally, species with similar niches would show concordant signals of isolation and divergence between refugia populations (Lessa et al. 2003; Soltis et al. 2006; Carnaval et al. 2009). There is clear evidence that taxa respond individualistically to climate change, but most of the responses of montane taxa appear not to be entirely independent due to cross-correlations among plant niches (Lessa et al. 2003; Heuertz et al. 2004; Hooghiemstra and Van der Hammen 2004; Williams et al. 2004). Ultimately, the genetic variation in refugia and the dispersal ability of each species would have a direct effect on the geographical distribution of genetic diversity of extant populations and thus affect the power to detect phylogeographical concordance among species (Heuertz et al. 2004; Excoffier et al. 2009; Sommer and Zachos 2009).

### The moist forests model

The moist forests hypothesis contends that changes in precipitation had a minor effect on the continuity of forest cover (Colinvaux et al. 2000; Bush and Silman 2004; Bush and de Oliveira 2006; Bush et al. 2009). The unchanging humidity conditions favored down-slope range expansion, little to no demographic growth, and population connectivity during the cold glacial periods, followed by the fragmentation into high altitude populations during warm interglacials (Fig. 2) (Ledig et al. 2002; Lessa et al. 2003; Jaramillo-Correa et al. 2008; Hensen et al. 2011; Schoville et al. 2011).

The sorting of lineages and demographic bottlenecks would be less likely to occur in continuous and large populations (Sommer and Zachos 2009). Although gene flow would homogenize genetic variation in an expanded moist forest, it is likely that divergent lineages pre-dating the last glacial would be present in extant populations and show a marked differentiation (Avise and Walker 1998). However, contrary to the refugia model, the divergent lineages would have an overall wide distribution and show no geographic structuring.

In general, genetic diversity would be favored and preserved throughout the species range by habitat heterogeneity and significant gene flow between populations. After fragmentation, extant populations would be composed of basically the same set of common alleles, resulting in a diffuse genetic structure (Caetano et al. 2008; Sommer and Zachos 2009; Schoville et al. 2011). The extended populations would be composed of several common alleles that would show a more complex genealogy than expected under the dry refugia model. The distribution of allele differences (mismatch distribution) would not fit a unimodal distribution (Rogers and Harpending 1992). In addition, as expected by mutation, rare alleles would be randomly produced over the species range and thus have low probability of clustering (Comps et al. 2001).

Under the moist forests model, the re-colonization process would basically consist of geographically localized altitudinal migration. In this respect, repeated founder effects are less likely to occur in up-slope mountain colonization because postfounding immigration would be highly probable (Hewitt 2004; Ehrich et al 2007; Hensen et al. 2011). Thus, species with prevailing altitudinal migration would not show any clear geographical gradient of genetic diversity (Tremetsberger et al. 2003). On the other hand, the connectivity between populations would result in the development of a pattern of isolation by distance (Slatkin 1993). This kind of pattern arises with geographically restricted dispersal, resulting in the accumulation of local genetic differences and an increase in genetic differentiation with distance.

## Genetic consequences

We propose that the climatic history of Neotropical cloud forests can be investigated by some of the basic genetic and demographic consequences of the dry refugia and moist forests models (Table 2). In order to successfully assess these models, the proposed consequences represent minimal requirements that can be easily tested with the classic tools of population genetics.

We acknowledge that the above-mentioned consequences do not represent the only testable outcomes of the range dynamics of species during the last glacial period. One possible differing consequence of the two models would be the timing of divergence between lineages. Yet, no models of divergence during glacial maxima have been developed specifically for cloud forest species. A good starting hypothesis would be that under the dry refugia model, divergence events for most loci would trace back to the LGM. However, given the different dynamics of molecular evolution across species, we believe is best to test the refugia hypothesis in terms of the demographic events accompanying the glacial cycles.

In any case, a multi-disciplinary approach including paleodistributional or paleoclimatological data would be more fruitful. Thus, the application of more sophisticated theoretical and analytical tools would be useful in testing the two models (Bayesian and coalescent inference: Ho and Shapiro 2011; colonization simulations: Bialozyt et al. 2006; paleodistribution and spatial modeling: Chan et al. 2011).

Traditionally, uniparentally inherited DNA (chloroplast, mitochondria) has been used to describe geographical patterns of genetic diversity. Nevertheless, the use of multiple genetic markers from different genomes would be advantageous to evaluate distinct attributes of the genetic structure and diversity of populations. In all cases, marker resolution would be the limiting factor when studying the demographic history of species. The choice of markers should be done bearing in mind relevant attributes of the molecular dynamics of distinct markers in particular taxa (e.g., the rate of mutation, the mode of inheritance, neutrality).

## Cloud forest phylogeography: testing the models

For the cloud forests, still there are virtually no genetic data for testing the above predictions (Ornelas et al. 2010; Gutiérrez-Rodríguez et al. 2011). Moreover, there are only a handful studies that directly test the genetic proposals of the refugia theory in Neotropical montane taxa. The next section briefly reviews some phylogeographic studies in order to highlight the lack of consensus regarding the glacial dynamics of Neotropical montane species (Table 3) (Velo-Antón et al. 2007; Caetano et al. 2008; Carnaval and Moritz 2008; Carnaval et al. 2009; Barker et al. 2011; de Mello-Martins 2011; Twyford et al. 2012).

**Table 3:** Supported model of demographic and genetic dynamics during the Last Glacial Maximum for the studied cloud forest and montane species in the Neotropics. (cpDNA = chloroplast DNA sequences, mtDNA = mitochondrial DNA sequences, nDNA = nuclear DNA microsatellites)

Region	Species	Model
*Mexico and upper Central America*		
	*Podocarpus matudae* (cpDNA) (Ornelas et al. 2010)	Moist forest
	*Palicourea padifolia (cpDNA)* (Gutiérrez-Rodríguez et al. 2011)	Dry refugia
	*Begonia heracleifolia* (cpDNA) (Twyford et al. 2012)	Moist forest
*The Antilles (Puerto Rico)*		
	*Eleutherodactylus portoricensis* (mtDNA, nDNA) (Barker et al. 2011)	Dry refugia
	*E. coqui* (mtDNA) (Velo-Antón et al. 2007)	Dry refugia
*Brazilian Atlantic forests*		
	Vertebrates (mtDNA, distribution modeling) (Carnaval and Moritz 2008)	Ambiguous
	*Hypsiboas* spp. (mtDNA, distribution modeling) (Carnaval et al. 2009)	Dry refugia

Ornelas et al. (2010) analyzed the phylogeographical structure of chloroplast DNA (cpDNA) haplotypes of *Podocarpus matudae* (Podocarpaceae) in cloud forests of Mexico and upper Central America. Their results appear to support the dry refugia hypothesis and its demographic consequences. The authors found a high genetic differentiation among populations and several common alleles with widespread distributions in distinct regions. In this context, the different common alleles are present in populations located around Toledo's (1982) proposed refugia. However, there is no evidence of range expansions, except in one population, and the separation of populations pre-dates the LGM.

In contrast, Gutiérrez-Rodríguez et al. (2011) explicitly tested the existence of refugia against a scenario of more continuous forest cover during the LGM. They used data on cpDNA haplotypes of *Palicourea padifolia* (Rubiaceae), a species inhabiting cloud forests in Mexico and upper Central America. There was significantly high population differentiation between populations within Toledo's (1982) refugia. The authors also found evidence of demographic expansions in distinct lineages. Thus, Gutiérrez-Rodríguez et al. (2011) support a history of rapid population growth from ancestral populations with small size. However, the authors do not support Toledo's forest refugia for the region and instead advocate in favor of more widespread refugial populations. The authors do not use paleoecological data and thus it remains unclear whether their results fully reject the dry refugia model or only contradict the geographical location and extent of the proposed refugia.

Twyford et al. (2012) analyzed the genetic structure of two species of *Begonia* (*B. heracleifolia* and *B. nelumbiifolia*, Begoniaceae) in Mexico and upper Central America. *B. nelumbiifolia* is a moist-adapted species living in altitudes from 70 to 1200 m, whereas *B. heracleifolia* is adapted to more seasonal conditions with an altitude range of 300-1700 m (Twyford et al. 2012). Populations of the latter species are characterized by a high genetic differentiation with significant isolation by distance. The authors tested for the genetic structuring of populations within the refugial regions proposed by Toledo (1982), but found that most of the variation was present within populations. Twyford et al. (2012) give no support to Toledo's refugia and instead propose the *in situ* survival of *B. heracleifolia*. In this respect, Twyford et al. (2012) argue that the monomorphic nature of *B. nelumbiifolia* is the result of population bottlenecks. Accordingly, their results appear to fit the history of expanding aridity during the LGM and partially support the existence of refugia, albeit not Toledo's refugia. However, the authors do not provide any demographic tests to corroborate their results and there is no reference to paleoecological data.

Barker et al. (2011) analyzed the genetic structure of the frog *Eleutherodactylus portoricensis*, a species living in cool-moist forests above 600 m in Puerto Rico. The authors found high genetic differentiation and reciprocal monophyly between mitochondrial (mtDNA) and nuclear DNA (nDNA) lineages occupying separate mountain ranges. In this respect, the authors claim that populations from distinct mountain ranges were continually isolated during cold periods, including the LGM, as a result of enhanced dryness. The authors performed several demographic tests with inconsistent results, but in general there are no signs of population changes during the last glacial period. In conclusion, Barker et al. (2011) support that populations of *E. portoricensis* have a long-history of isolation prompted by the enhanced glacial aridity recorded for the Caribbean region (González et al. 2008).

These results are comparable to those of Velo-Antón et al. (2007). These authors analyzed the genetic structure of the frog *E. coqui* in Puerto Rico using mtDNA, a species that inhabits a wide variety of habitats and occupies the complete altitude continuum (0–1300 m, Velo-Antón et al. 2007). As in *E. portoricensis*, populations of *E. coqui* are characterized by a deep genetic differentiation and reciprocal monophyly between lineages from distinct mountain ranges. In this respect, Velo-Antón et al. (2007) observed a unimodal mismatch distribution that, together with other demographic analyses, showed a history of sudden population decline and subsequent demographic expansion over the last 30 000 years.

Carnaval and Moritz (2008) and Carnaval et al. (2009) explicitly tested the dry refugia hypothesis in the Atlantic rain forests of Brazil by implementing paleodistribution modeling of the Atlantic Forest biome to infer climatically stable areas (refugia) since the LGM. Carnaval and Moritz (2008) then compared the location of the refugial areas with previous mtDNA sequence data for several vertebrate taxa from the Atlantic forest: opossums (*Marmosops incanus*; *Metachirus nudicaudatus*), the Atlantic tree rat (*Phyllomis pattoni*), lizards (*Gymnodactylus darwinii*), and the three-toed sloth (*Bradypus variegatus*). In the case of *B. variegatus* and *G. darwinii*, the authors observed higher levels of genetic diversity in inferred refugial populations than in populations outside refugia. In addition, these two species showed evidence of population expansions in accordance with the postulated history of refugia. Carnaval and Moritz (2008) did not find congruence between genetic and paleoecological data in all species. On the contrary, Carnaval et al. (2009) did find congruence between the inferred region of habitat stability and mtDNA sequence variation in three frog species: *Hypsiboas albomarginans*, *H. semilineatus* and *H. faber*.

These results are further supported by de Mello-Martins (2011), who used genetic data on vertebrates from the Atlantic forests of Brazil: howler monkey (*Alouatta guariba*), pitivers (*Bothrops*), the lesser wood creeper (*Xyphorynchus fuscus*), vampire bat (*Desmodus rotundus*), and frogs (*Hypsiboas*; *Rhinella crucifer*). Accordingly, de Mello-Martins (2011) inferred a more constant demographic history of the refugia populations compared with the recently colonized southern populations. Thus, at least for some species of the forests of the Brazilian Atlantic coast, the genetic consequences of refugia clearly agree with the paleoecological data and support the expansion of arid conditions over the LGM.

The direct test of the refugia theory has also been applied for seasonally dry tropical forests (Caetano et al. 2008; Poelchau and Hamrick 2011). Contrary to the case of cloud forests, the dryer conditions lead to a more continuous distribution of dry tropical forests during the LGM (Prado and Gibbs 1993; Pennington et al. 2009). In this context, Caetano et al. (2008) analyzed the populations of *Astronium urundeuva* (Anacardiaceae) in eastern South America, a species confined to seasonally dry tropical forests (Caetano et al. 2008). Based on cpDNA and nDNA, the authors inferred the existence of three groups of populations (southeast, central, and northwest) with a high genetic differentiation. The two extreme groups showed a positive correlation between genetic and geographical distance, indicative of isolation by distance. On the contrary, the central group showed a sign of admixture and did not have any spatial genetic structure. The authors inferred a range expansion from the two extreme groups into the central region and thus supported the more continuous distribution of these forests during dry glacial periods.

## Summary of the genetic patterns

It is clear that further genetic analyses are needed to establish the validity of the refugia hypothesis for cloud forests species. The inconsistency between studies can be the result of a differential response of species to climate change. The congruence among the inferred glacial dynamics of different species may be dependent on their distribution, ecology, or phylogenetic relationship. However, this inconsistency can also be the result of the difference in the analytical framework of distinct studies.

In this respect, the few molecular studies show little refinement in explicitly testing the genetic expectations of the dry refugia model and thus provide ambiguous evidence regarding the effect of widespread aridity on cloud forest taxa. Most of these studies do not incorporate available paleoecological data in their discussions. So far, the most detailed tests of the dry refugia model have relied on the direct comparison of the geographical location of proposed refugia and genetic data. Although valuable, the direct geographical inspection of proposed refugia is by itself inconclusive because these proposals are based on limited and inaccurate paleoecological data. This highlights the difficulty in pinpointing the exact location of former refugia without incorporating sufficient paleoecological data in phylogeographic studies.

The above discussion focuses on the genetic outcomes of the last glacial period and in doing so assumes that the genetic imprints of this period have overridden the effects of past glaciations. As observed in these studies, it is highly probable that the genetic divergence of many species pre-dates the last glacial period. However, the demographic events accompanying the LGM would have affected the distribution and frequency of mutations that occurred in the more distant past.

## Conclusions

The present study is not an exhaustive review of the literature. This review is intended to highlight the need of a general framework for conducting future studies on Neotropical phylogeography (Fig. 3). Depending on the geographical location and ecology of species, we strongly recommend a case-by-case examination of more detailed paleoecological, geographical, and ecological aspects of each species. The present review highlights the need for more detailed multi-disciplinary investigations to disentangle the climatic history of Neotropical forest communities. Most importantly, we strongly support the use of explicit alternative hypothesis in the statistical evaluation of the phylogeographic history of Neotropical forest species (Knowles 2001; Avise 2009; Hu et al. 2009; Hickerson et al. 2010; Brown and Knowles 2012; Dawson 2012). The genetic outcomes and hypotheses herein discussed represent a good starting point to achieve this purpose.

The climatic history of the Neotropics might be more complicated than stated herein. We are aware that other hypotheses might be more fitting to the data (e.g., microrefugia; Rull 2009) and that the alternative scenarios might not be mutually exclusive. The dry refugia and moist forests models are valid first approximations that are prone to further modification. Ultimately, there would be a continuum of species responses to climate change lying in between these two extremes, with particular patterns of genetic diversity being dependent on particular biological and historical attributes of species.

Further genetic studies should deal with co-distributed species, in particular those living within ecologically narrow forest communities, such as cloud forests. This might prove useful in uncovering general ecological response patterns of species to climate change, particularly precipitation. There is need of detailed molecular studies of Neotropical cloud forest taxa that include species with distinct ecologies and that represent different lineages. A first requirement would be to undertake broad scale latitudinal and altitudinal surveys and test them against paleoecological data. This can provide evidence of multiple refugial lineages and diversity gradients. If there is evidence of refugia, studies on the genetic structure can be conducted on finer spatial scales to evaluate the location and extent of these forest refugia. On a wider level, comparative research across species from different biomes, such as seasonally dry forests and deserts, is needed to determine the universality of the refugia hypothesis.

## Biographies

Luis E. Eguiarte is Professor and Head of the Evolutionary Ecology Department at the Institute of Ecology of the National Autonomous University of Mexico (UNAM). His current research projects include the study of the evolution, ecology, and conservation genetics of several groups of Neotropical plants, having significant contributions on these subjects.

Santiago Ramírez Barahona is a PhD student under the advisory of LEE and is mainly interested in the ecology, biogeography, and evolution of cloud forest species in the Neotropics.
